# Prevalence of Taurodontism in the United Arab Emirates: A Retrospective Study with a Global Comparison

**DOI:** 10.1055/s-0045-1811600

**Published:** 2025-09-11

**Authors:** Sangeetha Narasimhan, Shishir R. Shetty, Vinayak Kamath, Hiba S. Al-Daghestani, Vellore K. Gopinath

**Affiliations:** 1 Department of Oral and Craniofacial Health Sciences, College of Dental Medicine, University of Sharjah, Sharjah, United Arab Emirates; 2 Department of Orthodontics, Pediatric and Community Dentistry, College of Dental Medicine, University of Sharjah, Sharjah, United Arab Emirates; 3 Department of Public Health Dentistry, Goa Dental College and Hospital, Bambolim, Goa, India

**Keywords:** taurodontism, taurodont, dental anomalies, prevalence, enlarged pulp chamber, bull-like teeth

## Abstract

**Objective:**

Taurodontism is a developmental dental anomaly that can significantly impact various dental treatment procedures. This study retrospectively investigated the prevalence of taurodontism in the United Arab Emirates (UAE) and compared it with global prevalence rates.

**Materials and Methods:**

A total of 1,355 panoramic radiographs were retrospectively examined to identify cases of taurodontism. An extensive review of the literature was performed across three databases to identify studies reporting the global prevalence of taurodontism.

**Statistical Analysis:**

The global prevalence data were compared with findings from the UAE using Fisher's exact test or chi-square test.

**Results:**

The prevalence of taurodontism in Sharjah (UAE) was 0.66%. It was observed three times more frequently in the mandible (73.3%) than in the maxilla (26.7%). The mandibular second molar (46.7%) was the most affected tooth. Hypotaurodontism (66.7%) was the most prevalent type identified in the study. The pooled prevalence of taurodontism in the UAE was 1.4%, which closely aligned with the average prevalence observed in Middle Eastern countries. Based on the retrieved literature, North America recorded the highest prevalence at 31.3%, whereas the Middle East had the lowest prevalence at 1.9%.

**Conclusion:**

Taurodontism is less prevalent in the UAE compared with other regions worldwide. Globally, the occurrence of taurodontism varies significantly, with the highest prevalence rates reported in Canada, China, and Brazil. These differences may be influenced by genetic and environmental factors, variations in diagnostic methodologies, sample sizes, and inconsistencies in the inclusion and exclusion criteria applied across studies.

## Introduction

Developmental anomalies of the teeth encompass a range of disorders characterized by abnormalities in their morphology, size, and number, which can significantly impact the tooth form and function. 1 Taurodontism is defined as “a variation in the internal morphology of the pulp chamber, characterized by the extension of the pulp chamber to the root area.” 2 Gorjanović-Kramberger first described this condition in 1908 from prehistoric human dental fossils recovered in Croatia. 3 The term “taurodontism” was later coined by Sir Arthur Keith in 1913, derived from the Latin and Greek words “ *tauro* ” and “ *dont* ” meaning “bull” and “tooth,” respectively, due to the resemblance of these teeth to those of a bull. 4

The etiology of taurodontism indicates a developmental disturbance in Hertwig's epithelial root sheath, which plays a critical role in root formation. Root development is a complex process regulated at the molecular level through interactions between epithelial and mesenchymal-derived tissues. Any disruption in the induction of the epithelial root sheath by the ectomesenchyme, or a failure in the epithelial response, can delay or alter root morphogenesis. 5 The primary cause of taurodontism is believed to be a failure of the horizontal invagination of Hertwig's epithelial root sheath at the appropriate level during root formation. 6 This defect may arise from genetic mutations, familial inheritance (either X-linked or autosomal dominant), environmental influences, or hormonal imbalances. 7 8 Taurodontism may occur as an isolated condition or in association with various genetic syndromes. 9 More than 25 syndromes have been linked to taurodontism, with Klinefelter's syndrome being the most frequently reported. Studies have indicated that between 12.5 and 88% of individuals with Klinefelter's syndrome exhibit taurodontic teeth. 10 11

The characteristic features of taurodontism include an abnormally enlarged pulp chamber, apical displacement of the root canal orifice and pulpal floor, absence of cervical constriction at the cementoenamel junction (CEJ), and a more apically positioned root furcation. 6 12 Since the affected teeth appear normal during oral examination, the diagnosis of taurodontism is made exclusively through radiographic assessment. 7 9 Clinically, the anomaly may affect a single tooth or multiple teeth and may manifest unilaterally or bilaterally in both the primary and permanent dentition. 3 4 5

Taurodontism is classified into three categories: hypo, meso, or hypertaurodontism, based on the extent of apical displacement of the pulp chamber floor. 10 This classification system, originally proposed by Shaw et al in 1928, was considered highly subjective and lacked precision. 13 Since then, several researchers, including Keene (1966), Bloomberg et al (1971), Feichtinger and Rossiwall (1977), and Seow and Lai (1989) have introduced alternative diagnostic criteria for taurodontism. 14 15 16 17 In 1978, Shifman and Chanannel proposed a more objective method known as Taurodontism Index (TI), which quantifies the vertical distance between the horizontal line connecting the cementoenamel junction (CEJ) and the floor of the pulp chamber. According to this index, taurodontism is diagnosed when this distance is equal to or greater than 2.5 mm. 18 Taurodontism can complicate a range of dental treatments, including endodontic therapy, extractions, and orthodontic tooth movement, thereby increasing the risk of these procedures. 19 Therefore, it is critical to recognize the specific anatomical features of this anomaly during treatment planning. An accurate and thorough diagnosis is essential for developing effective treatment strategies that minimize complications and ensure optimal clinical outcomes. 1 19

Reported prevalence rates of taurodontism in humans vary from 0.2 to 11.3%. 1 Variations in prevalence among different populations may be primarily attributed to racial and ethnic differences. However, inconsistency in the type of teeth examined, the imaging modalities employed, and variations in diagnostic criteria and interpretation may also contribute to discrepancies in reported prevalence across populations. 8 20

A recent meta-analysis reports that ∼11.8% of the general population presents with taurodontic teeth. 18 Therefore, in a dental practice screening 100 patients per week, there is a significant likelihood of encountering at least five individuals with one or more taurodontic tooth. 7 Given the frequency of this condition and the potential risks it poses during treatment, it is imperative for dentists to have a comprehensive understanding of taurodontism. Awareness of its prevalence within specific geographic regions is crucial for informed diagnosis and treatment planning. While recent studies in the UAE have focused on dental anomalies related to tooth number, 21 research specifically addressing the prevalence of taurodontism remains limited. Hence, this study aims to assess the prevalence of taurodontism in Sharjah, UAE, using panoramic radiographs and to compare the findings with global data.

## Materials and Methods

### Study Setting and Design

This retrospective study was conducted at the University Dental Hospital Sharjah (UDHS), UAE. The study included patients who visited UDHS as outpatients, seeking various dental treatments between January 2022 and December 2024. Ethical approval was obtained from the University of Sharjah Research Ethics Committee (REC-23-02-21-01-F).

### Sample Size Calculation

Based on the prevalence of taurodontism reported in previous literature, 22 the sample size was calculated using the formula: *N* = *Z* 2 *× p (* 1 − *p)/d2* , where *N* = sample size, *Z* = Z statistic for a level of confidence = 1.96, *p* = expected prevalence or proportion = 17% = 0.17, and *d* = precision = 2% = 0.02.

*N* = 3.84 * 0.17 * 0.83/(0.02 * 0.02) = 1,355 participants.

### Inclusion Criteria and Exclusion Criteria

Patients were initially screened using demographic data and clinical history obtained from dental records, prior to retrieving any radiographs. Patients with syndromes or cleft lip and palate were excluded at this preliminary stage. High-quality orthopantomograms (OPGs) of patients aged 16 years and above were included in the study. OPGs with poor image quality or artifacts were excluded. Additionally, patients with multiple missing molars, fractures, deep caries, root canal treatments, crowns, or bridges in molars, as well as those undergoing fixed orthodontic treatment, were considered excluded.

### Sampling Method and Data Collection

A systematic random sampling method was used to select the radiographs. OPG images along with patients' demographic details were retrieved from the patient record management system at UDHS. All OPGs were acquired using a Sirona Orthophos XG5 (Dentsply, Germany) X- ray machine, operating at 64 kVp (kilovolt peak), 8 mA (milliampere), with an exposure time of 9 seconds. All the radiographs were examined by two experienced dentists for the identification and classification of taurodontic molars, following the diagnostic criteria established by Shifman and Chanannel. 23 Three anatomical dimensions were measured directly on the digital panoramic radiographs using calibrated image analysis software.

- **Pulp chamber height—** Measured as the vertical distance from the deepest point of the pulp chamber roof to the highest point of the pulpal floor.
- **Total crown-root length** —Measured as the distance from the same deepest point of the pulp chamber roof to the apex of the longest root.
- **CEJ-to-floor distance—** Measured as the vertical distance from a line connecting the mesial and distal cementoenamel junctions (CEJs) to the highest point on the pulpal floor.

Using these measurements, the **Taurodontic Index (TI)** was calculated with the following formula:

TI = (pulp chamber height/crown-root length) × 100.

According to this method, a tooth is identified as taurodontic if the TI exceeds 20, and the CEJ-to-floor distance is greater than 2.5 mm. Based on the TI values, taurodontism was further classified as hypotaurodontism—TI between 20 and 30, mesotaurodontism—TI between 30 and 40, and hypertaurodontism—TI of 40 or more. 23

### Data Source and Literature Search

Prevalence studies related to taurodontism were retrieved from three online databases, including PubMed, EBSCOhost, and Ovid, using the search terms (((taurodontism) *and* (taurodont)) *and* (prevalence)). The search that was conducted up to January 27, 2025, yielded a total of 481 studies (PubMed—147, EBSCOhost—116, Cochrane Library—218) restricted to articles published in English. Titles and abstracts were independently assessed by two authors (S.N., H.S.A-D.) using established inclusion criteria. Full texts were reviewed for studies whose eligibility could not be determined during the initial screening. Furthermore, the references of the selected articles were reviewed to identify other prevalence studies related to taurodontism. After excluding the duplicate records, case reports, narrative, and systematic reviews, a total of 59 prevalence studies were included. Two authors (V.K.G., S.R.S.) extracted the data, including the type of radiological assessment, population studied, sample size, and prevalence of taurodontism according to type, gender, and jaw distribution. The pooled prevalence of individual countries and geographic regions was calculated based on the data.

### Statistical Analysis

The collected data were entered into a Microsoft Excel spreadsheet and analyzed using IBM SPSS Statistics, Version 22 (Armonk, NY: IBM Corp-USA). Categorical variables were presented as frequencies and percentages and compared using Fisher's exact test or chi-square test as appropriate. A *p* -value of less than 0.05 was considered statistically significant.

## Results

**Fig. 1:**
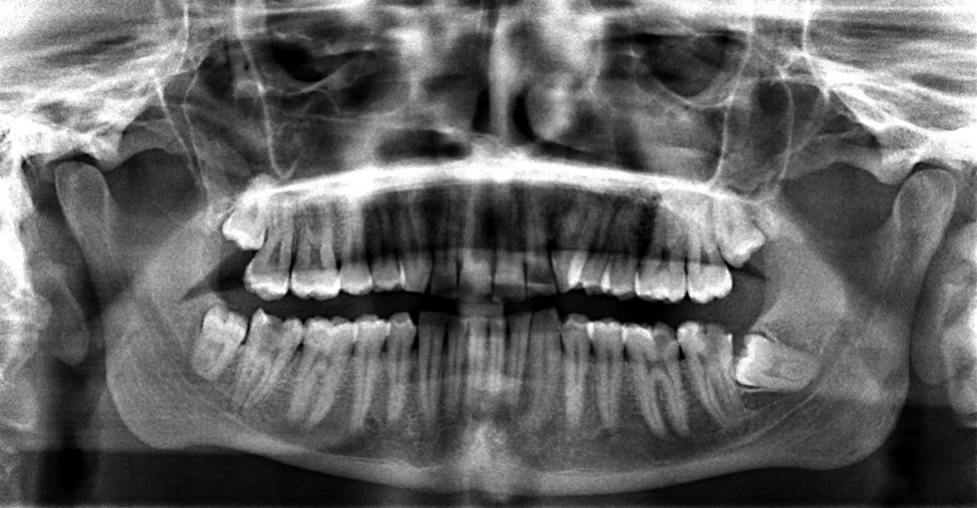
Orthopantomogram showing bilateral taurodonts in the maxillary teeth.

**Fig. 2:**
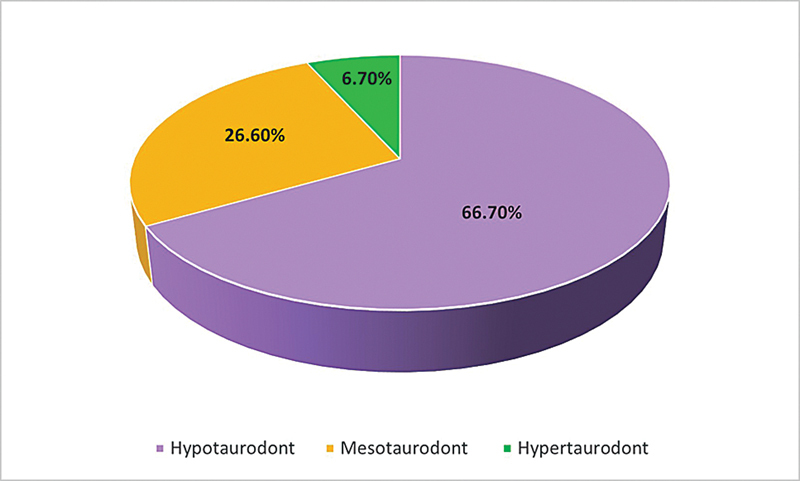
Distribution of types of taurodonts among the study samples.

The study participants comprised 857 men (63.2%) and 498 women (36.8%), ranging in age from 16 to 59 years (mean = 40.8 years, SD = 13.01). Nine out of the 1,355 study subjects exhibited taurodontism, yielding a prevalence of 0.66% in Sharjah (UAE). Table 1 presents the gender-based distribution of taurodont cases among the study population. Fig. 1 illustrates a representative OPG analyzed in the study. A total of 5 out of 497 women (1.0%) and 4 out of 853 men (0.47%) had at least one taurodontic tooth. Taurodontism was three times more common in the mandible (73.3%) than in the maxilla (26.7%). The mandibular second molar was the most affected tooth (46.7%), followed by the mandibular first molars (26.6%). Table 2 shows the distribution of taurodontic molars across the dental arches. Three out of the nine affected cases (33.4%) exhibited bilateral taurodontism. Among the 7,852 molars examined, 15 were diagnosed with taurodontism, representing a tooth-level prevalence of ∼0.19%. Based on arch-specific analysis, the maxillary prevalence was 0.11% (4/3,772), while the mandibular prevalence was 0.27% (11/4,080). Among the identified cases, 10 teeth (66.7%) were classified as hypotaurodonts, 4 (26.6%) as mesotaurodonts, and 1 (6.7%) as a hypertaurodont ( Fig. 2 ).

**Table 1:** Gender-based distribution of taurodonts among the study sample

Gender	Present	Absent	Total	%
Male	4	853	857	0.47
Female	5	493	498	1

**Table 2:** Distribution of taurodontic teeth in the maxilla and mandible

	**Maxilla**				**Mandible**				**Total**
Tooth	17	16	26	27	36	37	46	47	
No. of taurodont	1	1	1	1	2	4	2	3	15
Percentage	6.7	6.7	6.7	6.7	13.3	26.7	13.3	20	100

The literature search yielded 60 relevant studies, including two from the UAE, one conducted in the Emirate of Ajman and the other in Ras Al Khaimah. The majority of studies (34) were from Middle Eastern countries. Among West Asian nations, eight studies originated from India, three from China, and one from Thailand. Additionally, six studies were conducted in Europe, five in South America, two in North America, and one in Africa ( Table 3 ). Most studies utilized OPG or intraoral periapical (IOPA) radiographs to assess the prevalence of taurodontism, while only three employed cone beam computed tomography (CBCT). Based on the data obtained, taurodontic teeth were more frequently observed in the maxilla than in the mandible, with the maxillary second molars being the most frequently affected, followed by the mandibular second molars. Among the 60 studies reviewed, only 16 provided specific data on the types of taurodontism. Hypotaurodontism was reported as the most prevalent form, followed by mesotaurodontism and hypertaurodontism.

**Table 3:** Characteristics of the studies included in the global prevalence of taurodontism

Author	Year	Population	Age in yrs	Cases examined	Method	Prevalence (%)
Luke et al	2017	Middle Eastern countries	10–60	425	OPG	3.7
Zakaria et al	2018		17–60	400	OPG	1.25
Ruprecht et al	1987		NA	1,581	OPG	11.3
Afify	2012		12–30	878	OPG	0.1
Yassin	2016		5–12	1,252	OPG	1.4
Alassiry	2020		8–34	572	OPG	1.4
Jabali et al	2021		8–75	300	CBCT	8
ALHumaid et al	2021		7–65	1,104	OPG	0.1
Abdulrahman et al	2023		>18	385	OPG	2.1
Renugalakshmi et al	2023		5–17	1,442	OPG	1.25
AlHudaithi et al	2023		>12	384	OPG	2.6
Aldowsari et al	2024		6–14	1,987	OPG	1.66
Mallineni et al	2024		Mean age: 4.8	245	IOPA	2.8
Mahjoub et al	2024		8–27	923	OPG	1.8
Beshr	2018		8–40	1,560	OPG	0.6
Shoker et al	2023		6–12	2,583	OPG	1.16
Bronoosh et al	2012		15–61	510	OPG	5.5
Shokri et al	2014		7–35	1,649	OPG	3.34
Saberi	2016		>16	1,172	OPG	5.38
Jamshidi et al	2017		NA	2,360	OPG	22.9
Çolak et al	2013		15–50	6,912	OPG	0.26
Aren et al	2015		9–35	2,025	OPG	1.18
Simsek et al	2015		4–10	1,219	OPG	2.46
Citak et al	2016		12–25	1,964	OPG	1.93
Bilge et al	2018		6–40	1,200	OPG	4.41
Büyükgöze-Dindar	2022		12–60	43,880	OPG	0.1
Şenel et al	2023		NA	5,000	OPG	0.4
Shifman and Chanannel	1978		20–30	1,200	IOPA/BW	5.6
Einy et al	2022		9–29	624	OPG	33.5
Najm et al	2016		18–23	300	IOPA/OPG	0.3
Darwazeh et al	1998		18–78	875	IOPA	8
Alanzi et al	2024		8–12	546	OPG	6.6
Aldhorae et al	2019		9–52	1,202	OPG	0.91
Aboujaoude et al	2023		8–15	112	OPG	9.8
Frimpong et al	2024	Africa	NA	1,000	IOPA/OPG	17.1
Gupta et al	2011	West Asian countries	>14	1,123	OPG	2.49
Gupta and Saxena	2013		>18	1,360	IOPA	2.5
Patil et al	2013		13–38	4,143	OPG	0.4
Puttalingaiah et al	2014		>18	946	OPG	17.3
Bharthi et al	2015		>18	1,000	IOPA	2.8
Shah et al	2015		15–63	525	OPG	11.8
Harini and Don	2019		NA	14,022	OPG	0.17
Jain et al	2022		10–40	3,000	OPG	3.7
MacDonald-Jankowski and Li	1993		15–19	196	OPG	46.4
Li et al	2025		18–60	507	CBCT	22.29
Li et al	2023		18–67	580	CBCT	29.14
Pisek et al	2013		4–17	280	OPG	0.4
Goncalves-Filho	2014	South America	NA	478	OPG	27.19
Melo Filho et al	2015		Mean age: 14.2	300	OPG	23.3
Weckwerth et al	2016		>16	250	OPG	42.8
Pillai et al	2007		18–82	1,090	OPG/IOPA	11.28
Hoyte et al	2022		5–16	536	OPG	0.75
Baron et al	2018	Europe	< 18	551	OPG	15.06
Laganà et al	2017		8–12	4,706	OPG	0.04
Bürklein et al	2011		24–80	800	IOPA	2.25
Bäckman and Wahlin	2001		7	739	OPG/IOPA	0.3
Alt et al	2023		15–50	1,000	OPG	5.9
Drenski Balija et al	2022		12–16	506	OPG	1.2
Pach et al	2023	North America	NA	403	OPG	34
				251		27

Abbreviations: BW, bitewing radiograph; CBCT, cone beam computer tomography; IOPA, intraoral periapical radiograph; NA, not available; OPG, orthopantomogram.

(References of the Supplementary Material S1 , available in the online version only).

### Prevalence of Taurodontism in the Middle East

The overall prevalence of taurodontism in the UAE was calculated to be 1.4%, with women affected twice as often as men. Taurodontism was more frequently observed in the mandible (71.4%) compared with the maxilla (28.6%). The prevalence of taurodontism among the Middle Eastern countries ranged from 0.3 to 15.1%. The overall prevalence of taurodontism in the Middle East was found to be 1.9%, with a significantly higher occurrence among women compared with men ( *p* > 0.001) ( Supplementary Table S1 and Supplementary Fig. S1 [available in the online version only]).

### Global Prevalence of Taurodontism

**Fig. 3:**
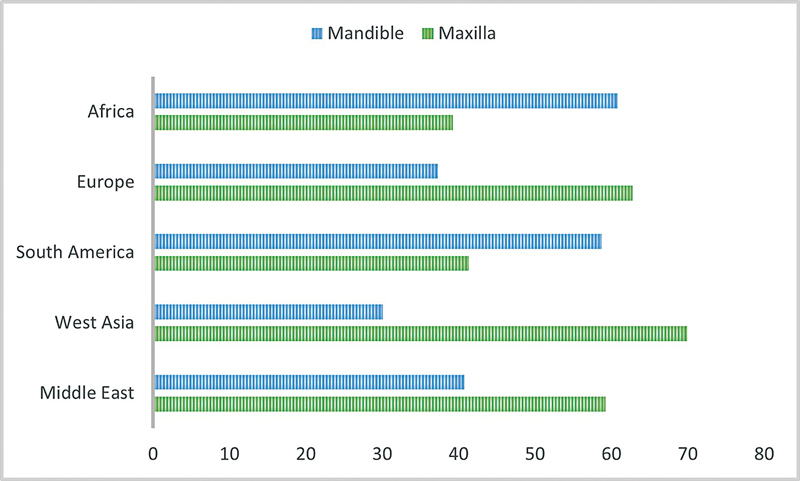
Arch-wise distribution of taurodonts among different regions of the globe.

The prevalence of taurodontism varies widely across global populations. Canada reports the highest prevalence at 31.3%, followed by Brazil (29.86%) and China (29.07%). In contrast, Italy records the lowest prevalence at just 0.04%. The pooled prevalence in India is 1.8%. Regionally, North America exhibits the highest prevalence (31.3%), followed by South America (16.4%). The cumulative prevalence in the Middle Eastern region (1.9%) closely aligns with that of Europe (2%). In West Asia (India, China, and Thailand), the prevalence stands at 3%, while Africa reports a prevalence of 17.1%. Gender-based and arch-wise distributions of taurodontism across different regions are summarized in Table 4 and illustrated in Fig. 3 .

**Table 4:** Prevalence of taurodontism across different regions of the world

	Overall		Male		Female	
	Prevalence (%)	*p* -Value	Prevalence (%)	*p* -Value	Prevalence (%)	*p* -Value
Middle East	1.9		4.3		3.8	
West Asia	3.0	< 0.001 a	2.7	<0.001 a	3.4	0.1
South America	16.4	< 0.001 a	13.9	<0.001 a	16.7	<0.001 a
Europe	2.0	0.36	1.6	<0.001 a	2.6	<0.001 a
North America	31.3	< 0.001 a	NA		NA	
Africa	17.1	< 0.001 a	NA		NA	

a *p* < 0.05, statistically significant; *p* > 0.05, nonsignificant.

## Discussion

Taurodontism is a developmental anomaly characterized by morphological alterations in tooth structure, resulting in an enlarged tooth body and shortened roots. 24 This study evaluated the frequency of taurodontism in the UAE and compared it with the global prevalence rates. The prevalence in Sharjah (UAE) was 0.66% which is lower than that reported in other Emirates, such as Ajman (3.7%) and Ras Al Khaima (RAK) (1.25%). 25 26 Despite methodological similarities, several factors may explain the variation in taurodontism prevalence rates between the Emirates. Our study had a larger sample size (1,355) compared with Ajman (425) and RAK (400). Larger samples yield more reliable prevalence estimates, while smaller ones are prone to variability and may misrepresent the true rates. The Ajman study included patients as young as 10 years old, while Sharjah and RAK included only individuals aged 16 and above. In younger individuals, developing roots may resemble enlarged pulp chambers, increasing the risk of misidentifying normal anatomy as taurodontism. Additionally, population heterogeneity across emirates may reflect variations in ethnic and genetic backgrounds. Finally, differences in examiner interpretation and diagnostic thresholds could further contribute to variability in detection and reporting.

In the present study, unilateral taurodontism (66.7%) was observed more frequently than bilateral cases (33.4%). However, existing literature suggests that bilateral taurodontism is generally more prevalent. 27 Interestingly, our findings reveal that taurodontism occurred three times more frequently in the mandible (73.3%), with the mandibular second molar being the most affected tooth. This contrasts with earlier studies, which typically reported a higher prevalence in the maxillary arch, particularly involving the maxillary second molar. 28 29 30 31 32 33 34 Furthermore, the current study results show that females were twice affected by taurodontism as often as males, aligning with previously reported data. 35 36 37 38 Nevertheless, a recent meta-analysis reported no meaningful correlation between gender and the prevalence of taurodontism. 18

The prevalence of taurodontism in UAE is relatively low (1.4%). This may be attributed to the limited number of studies conducted across the country. However, this figure closely aligns with the overall prevalence of taurodontism reported in the Middle Eastern Countries, which stands at 1.9%. Global data show a wide variation in the reported prevalence of taurodontism, ranging from 0.04 to 46.4%. 34 39 This considerable variability can be attributed to several factors, including genetic diversity, differences in diagnostic criteria, variation in sample sizes, and inconsistencies in inclusion and exclusion parameters across studies. For example, of the 12 studies conducted in Saudi Arabia, only two specifically examined Saudi nationals. Consequently, many studies report prevalence based on the geographical location of the research rather than the ethnic origin of the study population. In countries with large expatriate populations, such as those in the Middle East, the population often comprises individuals from diverse ethnic backgrounds. This lack of genetic homogeneity, combined with the absence of population classification based on ancestry, may introduce population bias, and potentially skew the accuracy of prevalence estimates for a specific country.

Another critical factor influencing the variability in reported prevalence rates is sample size. In the studies reviewed, sample sizes ranged from 112 to 43,880. According to Jamshidi et al, smaller sample sizes tend to yield less reliable prevalence estimates. To ensure accuracy and representativeness in taurodontism prevalence studies, a minimum sample size of 1,000 individuals is recommended. 29

The inclusion and exclusion applied in the taurodontism prevalence studies may also lead to substantial differences in reported rates. In contemporary populations, the prevalence of taurodontism varies depending on the specific group being studied. 3 For instance, the incidence is relatively high among individuals with certain syndromes, including cleft lip and palate. 40 41 The inclusion of syndromic patients in prevalence studies may significantly elevate the reported prevalence. Additionally, the results of certain prevalence studies that were conducted exclusively on children or orthodontic patients cannot be generalized to the broader population. 42 43 44 45 46

Taurodontic teeth are more prone to developing pulpitis and pulp necrosis, and their anchorage is often compromised. Dental procedures, such as extractions, restorations, and crown placements, can be challenging due to the altered tooth morphology and irregular root structure. 3 7 Clinical management of taurodontic teeth becomes particularly challenging during endodontic treatment because of factors such as the presence of additional canals, atypical canal orifice positioning, complex root canal configurations, and an increased incidence of pulp stones. Moreover, root canal therapy in taurodontic teeth is often accompanied by excessive bleeding, attributed to the larger volume of pulp tissue compared with normal teeth. Additionally, these teeth also demonstrate increased susceptibility to root resorption during orthodontic treatment. 7 10 Given the challenges in managing taurodontic teeth and their association with various syndromes, it is essential for dental practitioners to possess a comprehensive understanding of the condition's global and regional prevalence.

## Limitations of the Study

This study has certain limitations that should be considered when interpreting the results. One notable limitation is that the research was conducted within a single institution, which may restrict the generalizability of the findings to the broader population of the UAE. Additionally, the sample included both Emirati citizens and expatriates, reflecting the country's diverse demographic composition. However, due to the high proportion of expatriates, particularly those from Asian and Middle Eastern regions, in the present sample, the observed prevalence of taurodontism may not accurately represent its occurrence among UAE nationals alone. Our study, consistent with many previous epidemiological investigations, utilized OPGs as the standard diagnostic tool. However, being two-dimensional, they can produce image distortion and overlapping anatomical structures, which may lead to diagnostic inaccuracies. In contrast, CBCT provides high-resolution, 3D imaging that allows for more precise assessment of internal tooth anatomy, including root canal morphology and the vertical position of the pulpal floor, which are critical for accurately diagnosing taurodontism. Despite these advantages, CBCT was not utilized in our study due to its retrospective nature, which relied on existing radiographic records. Additionally, CBCT is not routinely prescribed for all patients because of its higher radiation exposure.

## Conclusion

This research suggests that the prevalence of taurodontism in the UAE is comparatively lower than in several other Middle Eastern countries and regions worldwide. The findings offer valuable insights for dental professionals globally, aiding in the assessment of taurodontism within their respective populations. Such understanding is essential for the accurate diagnosis and effective treatment of taurodontic teeth, which often pose challenges across various dental disciplines due to their increased likelihood of treatment-related complications.
