# Supplementary material for: Prevalence of Taurodontism in the United Arab Emirates: A Retrospective Study with a Global Comparison
Source: Eur J Dent. 2025 Sep 11;20(3):793–801. doi: 10.1055/s-0045-1811600 (PMC13337264; doi:10.1055/s-0045-1811600)
Supplement: Supplementary file 1 — Supplementary Material [file 10-1055-s-0045-1811600-s2544219.pdf]

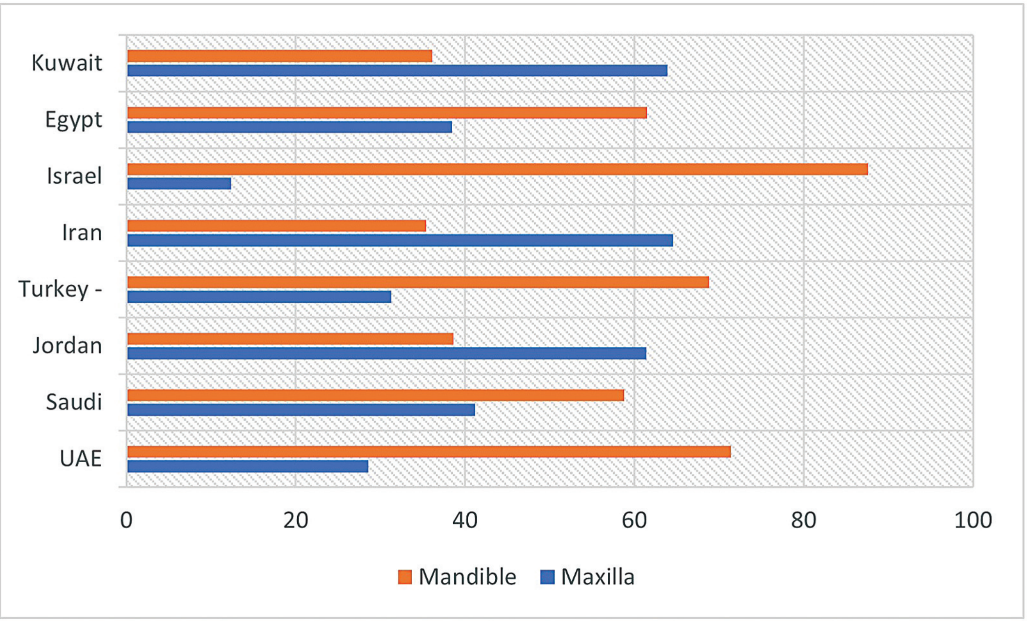

Supplementary Fig. S1 Arch-wise distribution of taurodonts among the Middle Eastern countries.

Supplementary Table S1 Gender-based prevalence of taurodontism in the Middle Eastern countries

	Overall		Male		Female	
	Prevalence (%)	*p*-Value	Prevalence (%)	*p*-Value	Prevalence (%)	*p*-Value
UAE	1.4		0.5		1.0	
Saudi	2.9	$<0.001^a$	3.5	$<0.001^a$	2.4	0.04^a
Jordan	8.0	$<0.001^a$	7.9	$<0.001^a$	8.1	$<0.001^a$
Turkey	0.4	$<0.001^a$	0.9	0.19	0.9	0.81
Iran	12.1	$<0.001^a$	17.5	$<0.001^a$	15.9	$<0.001^a$
Israel	15.2	$<0.001^a$	30.3	$<0.001^a$	37.1	$<0.001^a$
Egypt	0.9	0.11	1.2	0.08	0.7	0.57
Iraq	0.3	0.17	NA		NA	
Kuwait	6.6	$<0.001^a$	5.8	$<0.001^a$	7.3	$<0.001^a$
Yemen	10.9	0.24	0.6	0.71	1.1	0.81
Lebanon	9.8	$<0.001^a$	NA		NA	

[a] $p < 0.05$ statistically significant, $p > 0.05$ nonsignificant.

Supplementary Material S1: References For The Prevalence Studies Selected For This Article

1 Luke AM, Kassem RK, Dehghani SN, et al. Prevalence of dental developmental anomalies in patients attending a Faculty of Dentistry in Ajman, United Arab Emirates. Pesqui Bras Odontopediatria Clin Integr 2017;17(01):1–5
2 Zakaria H, Duarte C, Al Baloushi W. Prevalence of dental anomalies in patients from a teaching dental hospital in the UAE. Int J Orofac Res 2018;3(02):32–36
3 Ruprecht A, Batniji S, el-Neweihi E. The incidence of taurodontism in dental patients. Oral Surg Oral Med Oral Pathol 1987;63(06):743–747
4 Afify AR, Zawawi KH. The prevalence of dental anomalies in the Western region of Saudi Arabia. ISRN Dent 2012;2012(01):837270
5 Yassin SM. Prevalence and distribution of selected dental anomalies among saudi children in Abha, Saudi Arabia. J Clin Exp Dent 2016;8(05):e485–e490
6 Alassiry A. Prevalence and distribution of selected dental anomalies in Najran City of Saudi Arabia. Egypt Dent J 2020;66(03):1471–1482
7 Jabali AH, Chourasia HR, Wasli AS, et al. Taurodontism in maxillary and mandibular molars using cone beam computed tomography in a dental center in Saudi Arabia. Ann Saudi Med 2021;41(04):232–237
8 ALHumaid J, Buholayka M, Thapasum A, Alhareky M, Abdelsalam M, Bughsan A. Investigating prevalence of dental anomalies in Eastern Province of Saudi Arabia through digital orthopantomogram. Saudi J Biol Sci 2021;28(05):2900–2906
9 Abdulrahman BI, Aldahmash AM, Alghamdi HH, Alghamdi AH, Hamad TA, Ruished AF. Prevalence of dental anomalies among patients visiting the Riyadh Elm University clinics. Annals Dental Specialty 2023;11(02):40–45
10 Renugalakshmi A, Vinothkumar TS, Bokhari AM, et al. Prevalence of dental anomalies and its role in sex estimation among children of Jazan Region, Saudi Arabia. Children (Basel) 2023;10(04):759

 © 2025. The Author(s).

11 AlHudaithi FS, AlDuhayan NA, AlJohani LN, AlJohani SN, AlQarni HS, AlSawadi MH. Prevalence of dental anomalies among orthodontic patients: a retrospective study in Saudi Arabia. Cureus 2023;15(12):e49893
12 Aldowsari MK, Sulimany AM, Alkhathlan A, et al. Prevalence of dental anomalies in pediatric patients at King Saud University Dental Hospital, Riyadh, Saudi Arabia - a radiographic analysis. Children (Basel) 2024;12(01):13
13 Mallineni SK, Alassaf A, Almulhim B, Alghamdi S. Dental anomalies in primary dentition among Arabian children: a hospital-based study. Children (Basel) 2024;11(03):366
14 Mahjoub DT, Jarwan RK, Filimban LAZ, et al. The prevalence of dental anomalies among Saudi Population in Makkah, Saudi Arabia. J Orthod Sci 2024;13(01):46
15 Beshr KA, Mossa H. Prevalence of taurodontism in an Egyptian population permanent molar tooth. Egypt Dent J 2018; 64:4013–4017
16 Shoker NS, Metwally NM, Hadwa SM. Prevalence and distribution of diverse dental anomalies in an Egyptian children's population. Tanta Dent J 2023;20(02):111–117
17 Bronoosh P, Haghnegahdar A, Dehbozorgi M. Prevalence of taurodontism in premolars and molars in the South of Iran. J Dent Res Dent Clin Dent Prospect 2012;6(01):21–24
18 Shokri A, Poorolajal J, Khajeh S, Faramarzi F, Kahnamoui HM. Prevalence of dental anomalies among 7- to 35-year-old people in Hamadan, Iran in 2012-2013 as observed using panoramic radiographs. Imaging Sci Dent 2014;44(01):7–13
19 Saberi EA, Ebrahimipour S. Evaluation of developmental dental anomalies in digital panoramic radiographs in Southeast Iranian population. J Int Soc Prev Community Dent 2016;6(04):291–295
20 Jamshidi D, Tofangchiha M, Jafari Pozve N, Mohammadpour M, Nouri B, Hosseinzadeh K. Prevalence of taurodont molars in a selected Iranian adult population. Iran Endod J 2017;12(03): 282–287
21 Çolak H, Tan E, Bayraktar Y, Hamidi MM, Çolak T. Taurodontism in a central Anatolian population. Dent Res J (Isfahan) 2013;10(02): 260–263
22 Aren G, Güven Y, Guney Tolgay C, et al. The prevalence of dental anomalies in a Turkish population. J Istanb Univ Fac Dent 2015;49 (03):23–28
23 Simsek H, Bayrakdar I, Yasa Y, Cantekin K. Prevalence of taurodont primary teeth in Turkish children. Oral Health Dent Manag 2015; 14(01):23–26
24 Citak M, Cakici EB, Benkli YA, Cakici F, Bektas B, Buyuk SK. Dental anomalies in an orthodontic patient population with maxillary lateral incisor agenesis. Dental Press J Orthod 2016;21(06): 98–102
25 Bilge NH, Yeşiltepe S, Törenek Ağırman K, Çağlayan F, Bilge OM. Investigation of prevalence of dental anomalies by using digital panoramic radiographs. Folia Morphol (Warsz) 2018;77(02): 323–328
26 Büyükgöze-Dindar M, Tekbaş-Atay M. Prevalence of dental anomalies assessed using panoramic radiographs in a sample of the Turkish population. Chin J Dent Res 2022;25(03):189–196
27 Şenel ŞN, Erdem TL, Kendirci MY. Prevalence of dental anomalies in a group of Turkish people. Eurasia Dent Res 2023;1(01):1–6
28 Shifman A, Chanannel I. Prevalence of taurodontism found in radiographic dental examination of 1,200 young adult Israeli patients. Community Dent Oral Epidemiol 1978;6(04):200–203
29 Einy S, Yitzhaki IH, Cohen O, Smidt A, Zilberman U. Taurodontism —prevalence, extent, and clinical challenge in Ashkelon, Israel—a retrospective study. Appl Sci (Basel) 2022;12(03):1062
30 Najm AA, Mahdi AS, Al-Sudani RJ, Musa HH. Prevalence of dental anomalies among Iraqi dental students. J Bagh Coll Dent 2016; 325(3955):1–5
31 Darwazeh AM, Hamasha AA, Pillai K. Prevalence of taurodontism in Jordanian dental patients. Dentomaxillofac Radiol 1998;27 (03):163–165
32 Alanzi A, Bufersen N, Haider S, Abdulrahim M. Prevalence and distribution of dental anomalies in schoolchildren in Kuwait. Int Dent J 2024;74(03):566–572
33 Aldhorae KA, Altawili ZM, Assiry A, Alqadasi B, Al-Jawfi KA, Hwaiti H. Prevalence and distribution of dental anomalies among a sample of orthodontic and non-orthodontic patients: a retrospective study. J Int Oral Health 2019;11(05):309–317
34 Aboujaoude S, Rizk C, Sokhn S, Moukarzel C, Aoun G. Dental anomalies in a sample of Lebanese children: a retrospective study. Mater Sociomed 2023;35(04):319–324
35 Frimpong PB, Appau A, Ohene-Marfo E, Adu-Darko YA, Nartey NO. Prevalence of taurodontism in a tertiary hospital in Ghana. Open J Stomatol 2024;14(05):206–217
36 Gupta SK, Saxena P, Jain S, Jain D. Prevalence and distribution of selected developmental dental anomalies in an Indian population. J Oral Sci 2011;53(02):231–238
37 Gupta SK, Saxena P. Prevalence of taurodontism and its association with various oral conditions in an Indian population. Oral Health Prev Dent 2013;11(02):155–160
38 Patil S, Doni B, Kaswan S, Rahman F. Prevalence of taurodontism in the North Indian population. J Clin Exp Dent 2013;5(04):e179–e182
39 Puttalingaiah VD, Agarwal P, Miglani R, Gupta P, Sankaran A, Dube G. Assessing the association of taurodontism with numeric dentition anomalies in an adult central Indian population. J Nat Sci Biol Med 2014;5(02):429–433
40 Bharti R, Chandra A, Tikku AP, Arya D. Prevalence of taurodont molars in a North Indian population. Indian J Dent 2015;6(01): 27–31
41 Shah D, Garcha V, Garde J, Ekhande D. Prevalence of taurodontism among the patients visiting a dental teaching hospital in Pune, India: a retrospective orthopantomogram study. J Indian Assoc Public Health Dent 2015;13(01):83–86
42 Harini N, Don KR. Prevalence pattern of developmental anomalies of oral cavity in South Indian population - a hospital-based study. Drug Invent Today 2019;11(02):
43 Jain A, Sisodia S, Rana KS, Gupta C, Ansari I, Dholakia PP. The study of prevalence and distribution of shape anomalies of teeth in Indian population on the basis of age and gender. Cureus 2022;14 (08):e28532
44 MacDonald-Jankowski DS, Li TT. Taurodontism in a young adult Chinese population. Dentomaxillofac Radiol 1993;22(03):140–144
45 Li S, Min Z, Wang T, Hou B, Su Z, Zhang C. Prevalence and root canal morphology of taurodontism analyzed by cone-beam computed tomography in Northern China. BMC Oral Health 2025;25(01):5
46 Li Y, Qian F, Wang D, Wang Y, Wang W, Tian Y. Prevalence of taurodontism in individuals in Northwest China determined by cone-beam computed tomography images. Heliyon 2023;9(04): e15531
47 Wangsrimongkol T, Manosudprasit M, Pisek P, Chittiwatanapong N. Prevalence and types of dental anomaly in a Thai non-syndromic oral cleft sample. J Med Assoc Thai 2013;96(4, Suppl 4): S25–S35
48 Goncalves-Filho AJ, Moda LB, Oliveira RP, Ribeiro AL, Pinheiro JJ, Alver-Junior SR. Prevalence of dental anomalies on panoramic radiographs in a population of the state of Pará, Brazil. Indian J Dent Res 2014;25(05):648–652
49 Melo Filho MR, Nogueira dos Santos LA, Barbosa Martelli DR, et al. Taurodontism in patients with nonsyndromic cleft lip and palate in a Brazilian population: a case control evaluation with panoramic radiographs. Oral Surg Oral Med Oral Pathol Oral Radiol 2015;120(06):744–750
50 Weckwerth GM, Santos CF, Brozoski DT, et al. Taurodontism, root dilaceration, and tooth transposition: a radiographic study of a population with nonsyndromic cleft lip and/or palate. Cleft Palate Craniofac J 2016;53(04):404–412
51 Pillai KG, Scipio JE, Nayar K, Louis N. Prevalence of taurodontism in premolars among patients at a tertiary care institution in Trinidad. West Indian Med J 2007;56(04):368–371

 © 2025. The Author(s).

52 Hoyte T, Coppin E, Kowlessar A, et al. Prevalence of dental anomalies in Trinidad and Tobago. A retrospective study. Clin Investig Orthod 2022;81(02):117–125
53 Baron C, Houchmand-Cuny M, Enkel B, Lopez-Cazaux S. Prevalence of dental anomalies in French orthodontic patients: a retrospective study. Arch Pediatr 2018;25(07):426–430
54 Laganà G, Venza N, Borzabadi-Farahani A, Fabi F, Danesi C, Cozza P. Dental anomalies: prevalence and associations between them in a large sample of non-orthodontic subjects, a cross-sectional study. BMC Oral Health 2017;17(01):62
55 Bürklein S, Breuer D, Schäfer E. Prevalence of taurodont and pyramidal molars in a German population. J Endod 2011;37(02):158–162
56 Bäckman B, Wahlin YB. Variations in number and morphology of permanent teeth in 7-year-old Swedish children. Int J Paediatr Dent 2001;11(01):11–17
57 Alt KW, Wiesinger M, Nicklisch N. Prevalence of taurodontism in a modern Austrian samples. Bulletin of the International Association for Paleodontology. 2023;17(02):49–59
58 Drenski Balija N, Aurer B, Meštrović S, Lapter Varga M. Prevalence of dental anomalies in orthodontic patients. Acta Stomatologica Croatica. Int J Oral Sciences Dental Medicine 2022;56(01):61–68
59 Pach J, Regulski PA, Tomczyk J, Reymond J, Osipowicz K, Strużycka I. Prevalence of taurodontism in contemporary and historical populations from Radom: a biometric analysis of radiological data. J Clin Med 2023;12(18):5988

 © 2025. The Author(s).
